# Discerning the thermodynamic feasibility of the spontaneous coexistence of multiple functional vegetation groups

**DOI:** 10.1038/s41598-020-75050-4

**Published:** 2020-10-27

**Authors:** Meredith Richardson, Praveen Kumar

**Affiliations:** 1 Department of Civil and Environmental Engineering, University of Illinois at Urbana-Champaign, Urbana, 61801 USA; 2 Department of Atmospheric Sciences, University of Illinois at Urbana-Champaign, Urbana, 61801 USA

**Keywords:** Hydrology, Ecological modelling, Ecosystem ecology

## Abstract

Can the Second Law of Thermodynamics explain why ecosystems naturally organize into a complex structure composed of multiple vegetation species and functional groups? Ecosystem structure, which refers to the number and type of plant functional groups, is the result of self-organization, or the spontaneous emergence of order from random fluctuations. By considering ecosystems as open thermodynamic systems, we model and study these fluctuations of throughput signatures on short timescales to determine the drivers and characteristics of ecosystem structure. This diagnostic approach allows us to use fluxes of energy and entropy to calculate an ecosystem’s estimated work and understand the thermodynamic behavior of the system. We use a multi-layer canopy-root-soil model to calculate the energy and entropy fluxes of different scenarios for field sites across various climates. At each site, scenarios comprised of native individual plant functional groups and a coexisting multi-group composition scenario including all functional groups observed at the site are compared. Ecosystem-scale calculations demonstrate that entropy fluxes and work efficiency—the work performed for the amount of radiation entering the ecosystem—are greatest in the multi-group scenario when its leaf area is significantly larger than each of its individual functional groups. Thus, we conclude that ecosystems self-organize towards the vegetation structure with the greatest outgoing entropy flux and work efficiency, resulting in the coexistence of multiple functional groups and performing the maximum amount of work within the constraints of locally available energy, water, and nutrients.

## Introduction

Presence of vegetation on our planetary surface, when resources of water and nutrients are not limiting, is a ubiquitous feature. Often different plant species utilize niche space to create a plurality of simultaneous existence through competitive and/or symbiotic sharing of resources. The spontaneous emergence of such complexity across a range of climates suggests that this self-organization should be thermodynamically viable. We propose that thermodynamics can provide insights that can bolster scientific understanding of the coexistence of multiple vegetation species or functional groups within an ecosystem. By viewing ecosystems as open thermodynamic systems, we are able to calculate their entropy, which can allow us to identify possible thermodynamic drivers for the spontaneous emergence of complex vegetation structure.

The concept of viewing organisms and other natural phenomena as webs of open thermodynamic systems has been utilized across scientific disciplines for several decades. Prigogine^1,2^ and other chemists, physicists, and biologists studied and developed the ideas of open thermodynamic systems in the early twentieth century with the goal of conceptualizing the growing complexity of organisms and other biological systems. They discovered that this complexity comprises of a hierarchy of irreversible processes leading to systematic organization which maintains the system in a state far from thermodynamic equilibrium^1–6^. Soon the idea of system theory was extended to other natural systems including ecological and Earth systems^7–9^. It became understood that ecosystems can be categorized as open thermodynamic systems existing far from thermodynamic equilibrium maintained by the spatial imbalance of energy in the form of state variables, such as temperature or geopotential height^10,11^. Energy and mass naturally flow along gradients from high to low concentrations, and more rapid dissipation of these gradients is made possible by the formation of structures^9,10,12^. These so-called dissipative structures give the system a form of organization that emerges without external directive or predetermination, called self-organization^13^. This local self-organization results in low local entropy and greater overall system entropy due to the dissipation of the driving gradient and the decreasing spatial heterogeneity of the associated state variables^1,10^. Such dissipative structures spontaneously emerge through self-organization, which can be exemplified in Earth systems from convection cells on the global scale to vegetation on the local scale^10,11^.

Ecologists further expanded these ideas to understand the direction of ecosystem evolution and quantify the distance of a system from equilibrium^14–16^. Concepts such as exergy and eco-exergy have been fruitful for the understanding of relative stages of ecosystem development and developing thermodynamic principles of ecology, such as the irreversible nature of ecosystem processes and the increasing disequilibrium of ecosystems^17–22^. However, exact quantifications of exergy are not feasible due to the requirement of the knowledge of the equivalent ecosystem in equilibrium—similar to an ‘inorganic soup’^22^ with no lifeforms. Thus, when comparing differences in similar ecosystems based on the composition of functional groups, a new framework must be developed.

While growth and development of ecosystems has been studied from the thermodynamic perspective, the thermodynamic basis for the self-organization towards one dissipative structure over another remains unexplored. This paper seeks to fill this gap in understanding by comparing the thermodynamic behavior of different possible vegetation structures for a given ecosystem to identify if one results in a thermodynamic advantage over others.

We characterize ecosystem composition and vegetation structure in terms of the number and type of plant functional groups present. A plant functional group corresponds to a set of species that performs similar functions^23^. We accept the existence of an observed vegetation structure as a probabilistic, self-organized outcome. However, little has been done to compare the prospects of other vegetation scenarios that do not emerge. The probabilistic set of possible vegetation structures at a given site is based on the available energy, nutrients, and water. We aim to seek clarity on this topic by adding an additional parameter—thermodynamic advantage. We utilize work and entropy flux as metrics to compare thermodynamic behavior and determine if a certain vegetation structure has a thermodynamic advantage over others, thereby making it more probable. In classical mechanics, work is defined as the energy required for motion or the “the flow of heat” for heat engines in particular^13^; for ecosystems, we interpret this existing definition to estimate work as the sum of the latent and sensible heat leaving the system (i.e. the energy leaving the system through molecular motion of water vapor and air molecules). In these systems, self-organization in the form of vegetation emerges as a result of the heat dissipation throughout the vertical temperature gradient between the atmosphere at the top of the canopy and the soil-surface. Work is a measure of the ability of an ecosystem (by way of vegetation) to diminish this temperature gradient through the redistribution of heat. The entropy flux leaving an ecosystem—calculated from its temperature and outgoing energy flux—is a measure of the disorder of the outgoing energy, or the inability of this energy to perform work. However, high outgoing entropy flux does not always mean that more work has been performed. Longwave radiation has high entropy, but it is not a component of work; it is a form of radiative energy that is a passive response to the temperature state of its source and leaves the control volume without directly affecting the distribution of heat throughout the vertical profile. Thus, it is wasted energy. To distinguish between work and wasted energy, we introduce the concept of work efficiency as the work performed for the amount of radiation entering the ecosystem. Work efficiency measures an ecosystem’s ability to effectively dissipate the incoming energy throughout the ecosystem through conversion of energy into alternate forms. Ecosystems with greater work efficiency more effectively decrease the temperature gradient imposed on the ecosystem, giving the ecosystem a thermodynamic advantage. Since entropy flux and work efficiency are not equivalent, both metrics are important for the interpretation of thermodynamic advantage.

Our premise is that ecosystems with more plant functional groups—corresponding to more complex dissipative structures—produce more total entropy and perform more work, resulting in a higher work efficiency than ecosystems having only one plant functional group. This leads to our main research question: *Does the existence of multiple functional groups offer a thermodynamic advantage?*

To address this question, we model and compare the thermodynamic behavior of representative ecosystems consisting of multiple functional groups with that of hypothetical single-functional-group scenarios comprising of the individual native functional groups that make up the coexisting multiple functional groups. This is with the acknowledgement that the energy, water, and nutrients at each site support the existing functional groups. We do not alter the biomass or any additional parameters of these individual functional groups when modeled individually because we do not know how the energy, nutrients, and water of the ecosystem would support additional growth of these species if the others did not exist. Self-organization is non-linear, subject to chance outcomes to which we cannot predict how the alternative ecosystem would be structured. Thus, we compare the multi-group scenario with the known composition of the individual functional groups as they are observed to discern if there are advantages that promote the thermodynamic feasibility, or drivers towards the existing multi-group scenario. Using an open thermodynamic system framework and a 1-dimensional multi-layer canopy model (MLCan) which has been widely used and validated^24–30^, we simulate three climatologically-different natural ecosystems to determine the energy and entropy fluxes across the ecosystem control volume consisting of the canopy, roots, and soil (Fig. S1). Energy fluxes considered are shortwave and longwave radiation, and sensible and latent heat. The model calculates the energy and entropy fluxes in each layer over a 2-year study period, 2004–2005. These years were chosen based on continuous data availability in order to use the same study period across all study sites. Entropy flux, work, and work efficiency at each timestep (half-hourly or hourly) are then calculated as the net sum over all 21 layers (20 canopy layers and 1 layer for the ground surface) to complete the ecosystem-level analysis, providing a description of thermodynamic behavior that is able to capture the subtle differences among different simulation scenarios.

Three sites from the FLUXNET2015 dataset^31^ are modeled: Santa Rita Mesquite (SRM) in Arizona, Willow Creek (WCR) in Wisconsin, and Tapajos National Forest (TAP) in Pará, Brazil (Fig. S2)^32–34^. The SRM and WCR sites are modeled with two functional groups based on the composition of their dominant vegetation, and the TAP vegetation is divided into four functional groups (based on the details in Domingues et al.^35^). For simplicity, the functional groups are abbreviated as: understory (UN), mid-canopy trees (MT), overstory trees (OT), and lianas (L). The scenario with multiple functional groups is abbreviated as MG. Site-specific classifications can be found in Table S1 of the Supplementary Information. At each location we compare the existing scenario of vegetation consisting of multiple functional groups with hypothetical scenarios of each one of the individual functional groups present. For all scenarios, we calculate the entropy flux ($J_{eco}$; see Eq. 7), work (*W*; see Eq. 11), and work efficiency (*WE*; see Eq. 13). We define thermodynamic advantage as the production of larger entropy fluxes as well as greater work efficiency by the ecosystem as a whole. *We hypothesize that the multiple-functional-group systems are more thermodynamically advantageous than or similar to their respective single-group scenarios.*

## Results

**Figure 1:**
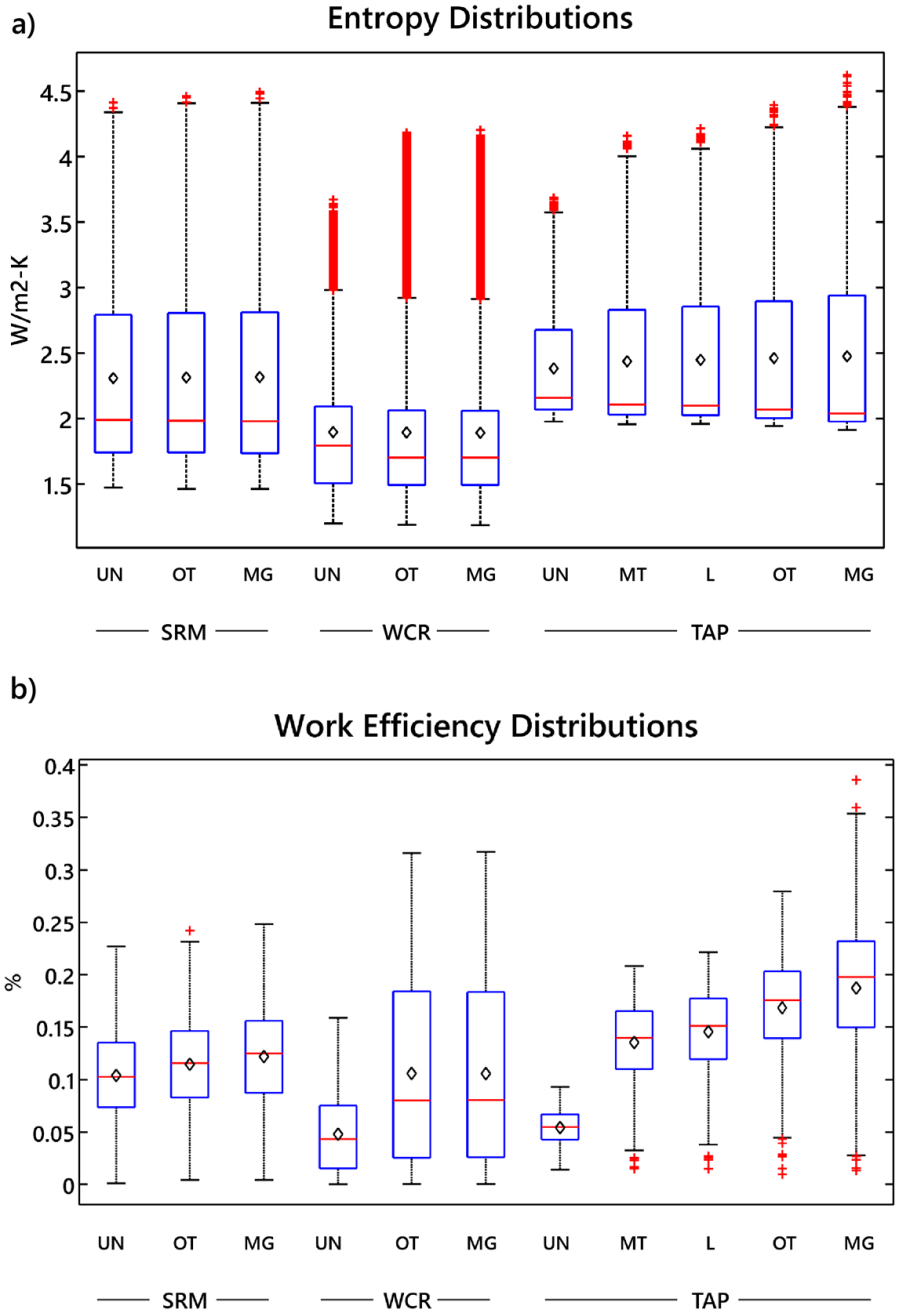
Entropy and work efficiency flux distributions. Illustration of the variability of (**a**) entropy flux, and (**b**) work efficiency associated with each functional group and coexisting multi-functional vegetation groups (see Table S1). The distributions of entropy fluxes are developed based on each time step of simulation for the 2-year study period, and the distributions of work efficiency are calculated based on daily energy fluxes (see Eq. 13). Means are shown as black diamonds.

The distributions of entropy fluxes at each timestep and the distributions of daily work efficiency for the 2-year study period are shown in Fig. 1. The three sites have different ranges of entropy fluxes and work efficiencies due to distinctions in the local availabilities of water, energy, and nutrients. WCR, SRM, and TAP have relatively energy-limited, water-limited, and nutrient-limited environments, respectively. Thus, the entropy and work efficiencies should not be directly compared across sites. However, within each site the ranges gradually change as we look across the different functional group scenarios. SRM has the least variability amongst its functional groups, and TAP has the most. Considering the entropy fluxes and work efficiencies, the multi-group scenarios all appear to have distributions similar to or consisting of larger values than the other scenarios for each site.

Using the Miller Jacknife and Kolmogorov–Smirnov tests, the distributions of entropy fluxes and work efficiency for the multiple functional group scenarios at each site are compared for statistically significant differences with each of their individual functional groups. Overall, the results indicate that multiple-functional-groups either have a thermodynamic advantage over single-groups or they are not at a disadvantage due to greater or similar values of entropy flux and work efficiency. There was a statistically significant difference in the distributions of entropy and work efficiency between the MG scenarios and each of the individual functional groups except for WCR-OT. This case is unique since WCR-UN only contributes to 3% of the total leaf area of WCR-MG, meaning that the WCR-OT and WCR-MG are very similar in vegetation composition. For all other cases, the values of entropy and work efficiency were significantly larger in the MG scenarios, indicating thermodynamic advantage. The “Statistical analysis” section in the “Methods” lays out the statistical tests and related analyses for comparing these entropy flux and work efficiency distributions.

### Factors impacting entropy flux

**Figure 2:**
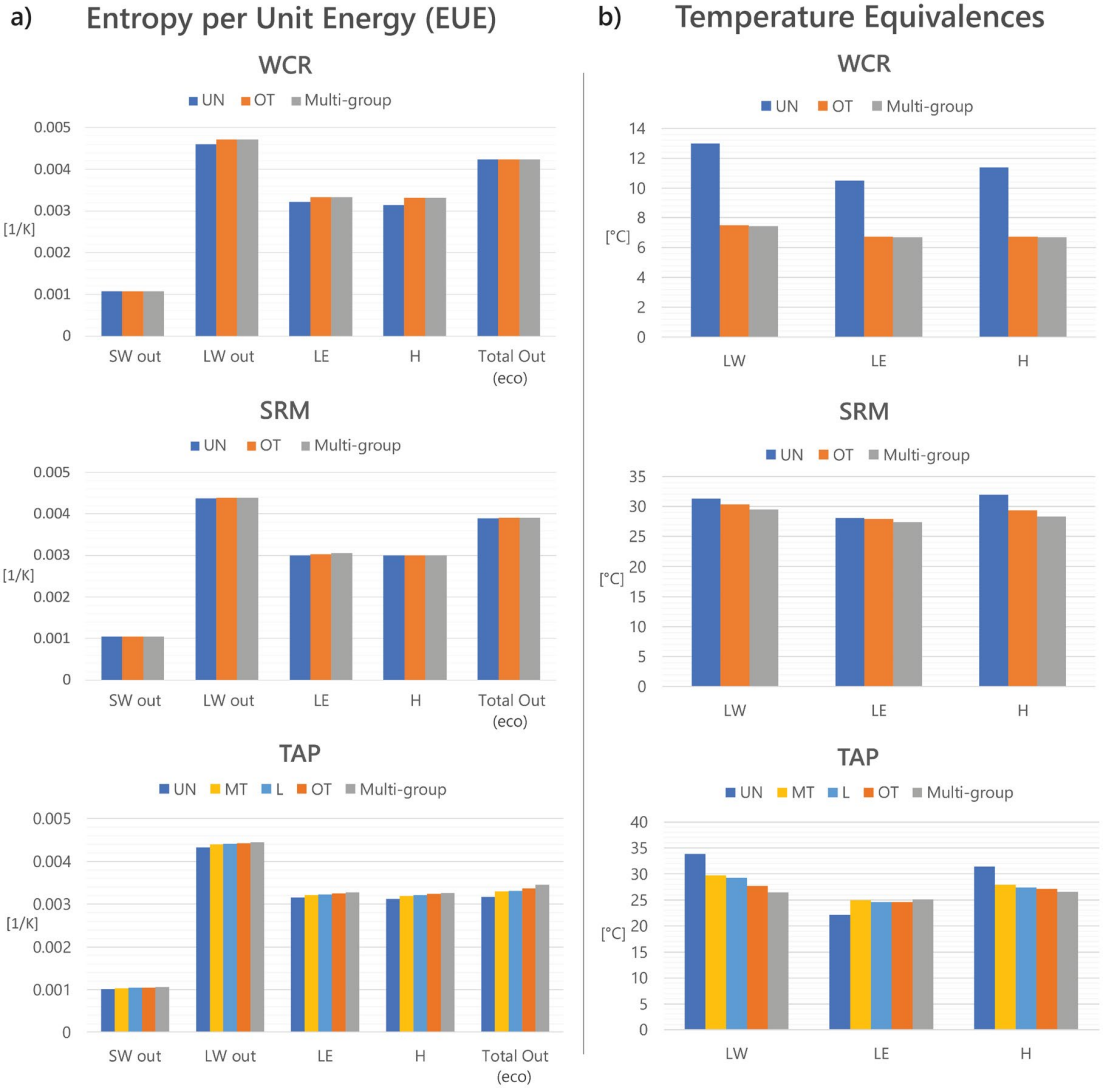
Entropy per unit energy and temperature equivalences. (**a**) Entropy per unit energy (*EUE*) by energy category for all sites (see Eq. 8). Colors refer to vegetation functional groups: *UN* understory, *MT* mid-trees, *OT* overstory trees, *L* lianas, and multi-group consisting of all functional types observed in the ecosystem. In general for each energy category, more *EUE* is associated with increasing leaf area index (LAI). (**b**) Temperature equivalences ($T_{eq}$) by energy category for all sites. In general for each energy category, temperatures are cooler with increasing LAI. Despite similar equivalent temperatures for emitted longwave radiation ($LW_{out}$), latent heat (*LE*), and sensible heat (*H*), $LW_{out}$ is by far the greatest contributor to $EUE_{eco}$ (see Eq. 9). [The weather forcing for all model simulations is the same across scenarios for each site].

The behavior of an ecosystem’s entropy is determined by the combined variation of its individual energy fluxes leaving the system, such as: shortwave radiation (*SW*), longwave radiation (*LW*), latent heat (*LE*), and sensible heat (*H*). For each scenario at each site, an entropy per unit energy (*EUE*) value is computed for all energy fluxes (see Eq. 8) and reported as an average over the simulation period in Fig. 2a. Each ecosystem has its own partitioning of energy among these categories, leading to differences in total entropy per unit energy leaving the ecosystem ($EUE_{eco}$), corresponding to “Total Out” in Fig. 2a.

The *EUE* for each energy category can be explained by the temperature of its source. *SW* originates from the sun, so its *EUE* is based on the temperature of the sun. However, outgoing longwave radiation ($LW_{out}$), *LE*, and *H* originate from the leaves as well as the soil surface. The total outgoing energy of the ecosystem is a resultant of the energy leaving each of the canopy layers including the soil surface within MLCan, each with its own temperature. Therefore, we calculate temperature equivalences ($T_{eq}$) for each of these energy categories based on the weighted average of the temperature of each leaf and soil layer contributing to the overall energy flux of that category (Fig. 2b, Eq. 5). Entropy is calculated directly from temperature (Table 1), leading to an important inverse relationship between *EUE* and $T_{eq}.$

Figure 2b shows that for each of the sites, across almost all energy categories the UN scenario has the highest $T_{eq}$ and the multi-group scenario has the lowest. Due to the inverse relationship between $T_{eq}$ and *EUE*, all of the *EUE* averages for each category in Fig. 2a are smallest for the UN scenarios and largest in the multi-group scenarios, though some of these differences are marginal. From this pattern, one would expect that this would lead to a clearly greater overall $EUE_{eco}$ for all multi-group scenarios (“Total Out” in Fig. 2a). Yet, this is not the case for all sites, as TAP is the only one in which the average $EUE_{eco}$ varies considerably among functional group scenarios.

Although *EUE* averages tend to increase with leaf area index (LAI) and when multiple functional groups coexist, each energy category has different relative values of *EUE*. Further, *EUE* can be interpreted as an indicator of how degraded a particular form of energy is and the ability of that energy to perform additional work. *SW* has the least *EUE* across all sites and scenarios (Fig. 2a), so it has the greatest capacity for work to be done; plants are able to use this energy to perform work. Yet, *SW* is radiative energy, so it does not perform work itself. On the other end, $LW_{out}$ has the highest *EUE* of the energy categories studied here, meaning it is the most degraded with little capacity for additional work to be performed from it. In the middle, *LE* and *H* are more degraded than *SW*, but they are still able to perform physical work in the ecosystem through convection and conduction and contribute to the redistribution of heat throughout the vertical profile of the ecosystem. Since we are only considering the fluxes that enter and leave the ecosystem control volume in this analysis and all irreversible work releases heat, *LE* and *H* are also proxies for the work performed within the ecosystem internally (i.e. by the vegetation itself). Overall, when an ecosystem has low thermodynamic efficiency and high $LW_{out}$, it degrades the incoming *SW* quickly without performing much work within the ecosystem and constitutes wasted energy. However, higher values of *LE* and *H* leaving the ecosystem mean that more work has been performed.

**Figure 3:**
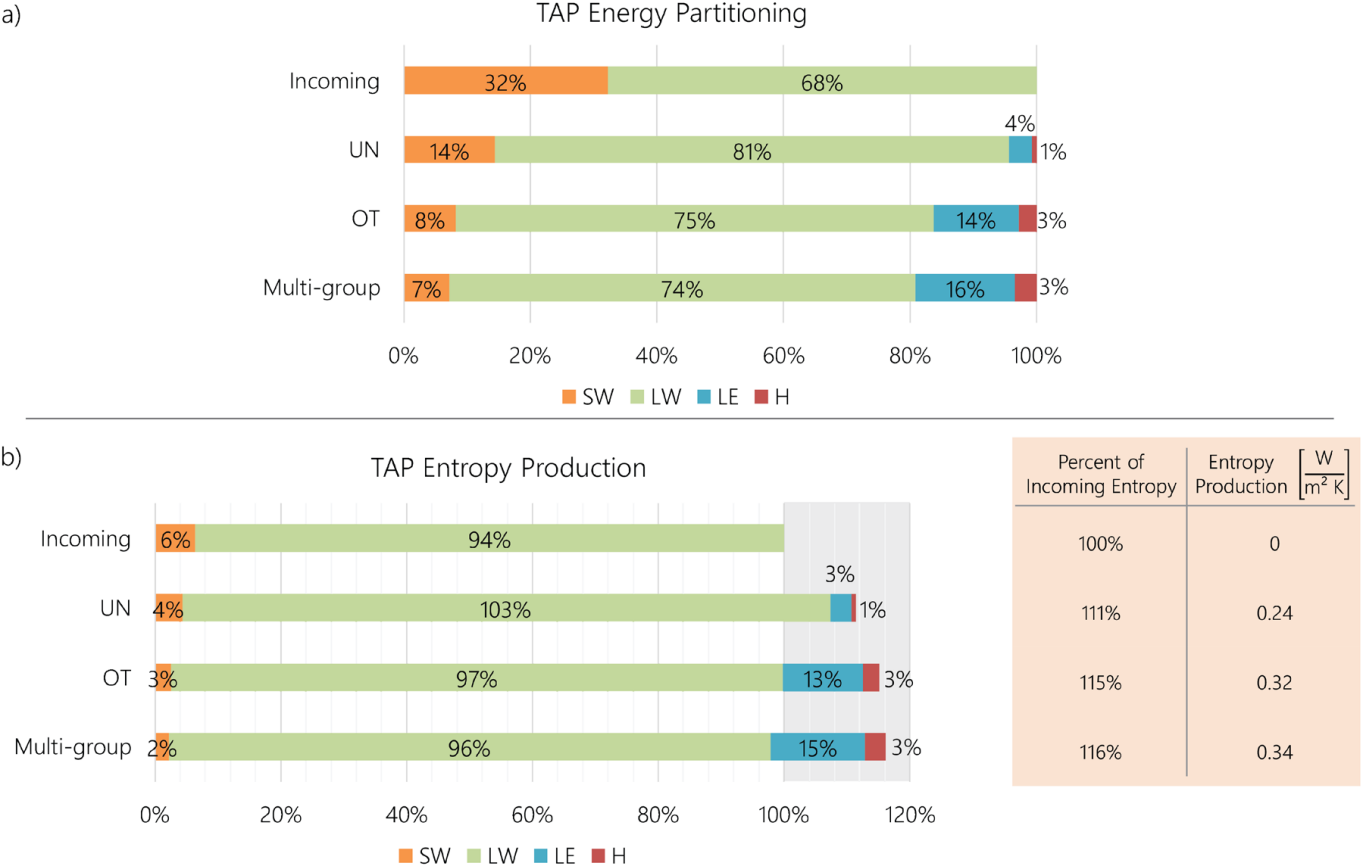
Energy and entropy partitioning by scenario for TAP. (**a**) Partitioning of incoming radiation into outgoing shortwave radiation (*SW*), longwave radiation (*LW*), latent heat (*LE*), and sensible heat (*H*) for three scenarios at TAP. From UN to OT to Multi-group, the percentage of $LW_{out}$ decreases as the sensible and latent heat increases. A larger partitioning towards $LW_{out}$ (i.e. UN) leads to greater weight towards a higher *EUE* value for an ecosystem’s dissipation efficiency (Fig. 2a). (**b**) The corresponding entropy flux for the incoming radiation and outgoing energy flux of three TAP scenarios presented as a percentage of the total incoming entropy flux. Unlike energy, entropy is not conserved, but produced—indicated by a percentage greater than 100 and shaded in grey. The table displays the percent of the scenarios’ outgoing entropy flux relative to the incoming entropy flux, indicating a 11–16% increase in entropy produced by the functional groups. Since *LW* has the largest *EUE* of all the energy categories (Fig. 2a), its percentages in (**b**) are larger than in (**a**).

Since each scenario partitions the incoming energy differently throughout the ecosystem, the considerably higher overall *EUE* for $LW_{out}$ has important implications for overall $EUE_{eco}$ and entropy fluxes, yielding larger values when more outgoing energy is allocated towards $LW_{out}$. Figure 3a displays the average partitioning of incoming radiation among the various energy categories at TAP, and Fig. 3b indicates the corresponding entropy fluxes presented as percentages of the incoming entropy flux and disaggregated into different energy categories. The energy and associated entropy entering the ecosystem are the same for all functional groups, but the outgoing fluxes vary significantly among them. Due to conservation of energy, the outgoing energy for all scenarios corresponds to 100% of the incoming radiation. Alternatively for entropy, this percentage is greater than 100%, indicating entropy production (shaded in grey). At TAP and similarly at the other two sites, the multi-group scenario produces more total entropy on average than the other two scenarios.

However, the contribution of each energy category towards the total entropy differs among functional groups. As LAI increases from UN to OT and with the addition of multiple functional groups, the proportion of *LE* and *H*, or work, increases while the outgoing radiation decreases (Fig. 3). The UN scenario partitions more energy towards $LW_{out}$ than the OT and multi-group scenarios. Since *LW* has the largest *EUE* of all the energy categories (Fig. 2a), its percentages in Fig. 3b are larger than in Fig. 3a. This observation, consistent across all sites, indicates that UN scenarios are able to make up for lower performing *EUE* values by partitioning more energy towards the higher entropy-producing energy category, $LW_{out}$. Thus, even though the UN scenario has the lowest *EUE* for all energy categories, its total $EUE_{eco}$—and overall ability to degrade the incoming *SW*—and entropy production are similar to the other scenarios since it has more outgoing energy partitioned towards the largest *EUE* category, $LW_{out}$. However, $EUE_{eco}$ is an indication of energy degradation, not work performed. Thus, the additional assessment of work and work efficiency is necessary for the interpretation of thermodynamic advantage.

### Work as an indicator of self-organization

**Figure 4:**
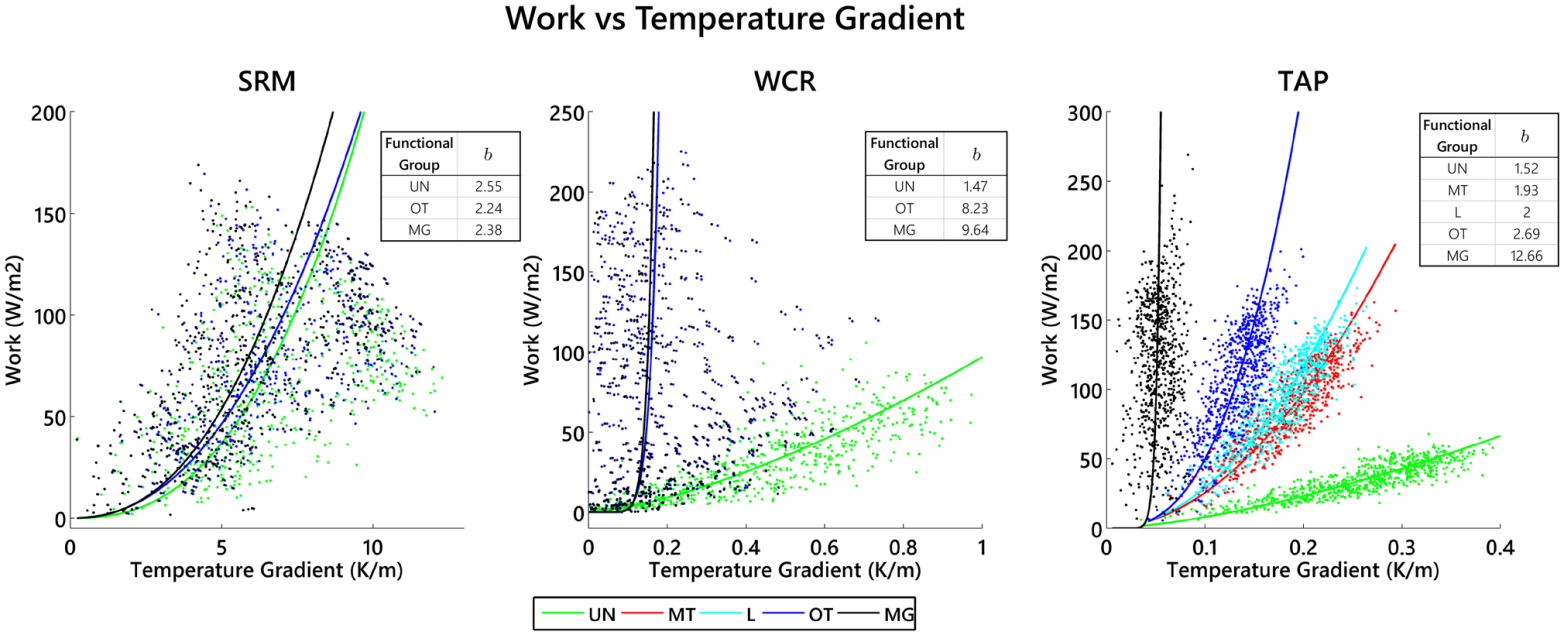
Plots of work versus temperature gradient for all functional groups and sites. Power functions are fit to the data: $W=a(\Delta T/\Delta z)^b$. Parameter *b* is reported in the upper right corner of each plot. For each site LAI increases in each functional group scenario from left to right in the legend at the bottom (i.e. UN has the smallest LAI and MG has the largest—see Fig. S2 in the Supplementary Information). Trends in *b* indicate that power increases with LAI among functional groups and is the largest in the MG scenario for all sites except the SRM-UN scenario. A few values corresponding to negative temperature gradients are removed from the WCR scenario since they are a function of the presence of snow rather than self-organization.

As discussed in the previous section, partitioning of energy and entropy fluxes are important for understanding the overall thermodynamic behavior of ecosystems. Energy fluxes with large *EUE* values result in greater entropy production for an ecosystem but do not always yield more work performed. Work—estimated as the sum of the ecosystem’s latent and sensible heat fluxes (*W*; Eq. 11)—represents the ability of an ecosystem to diminish the temperature gradient through the ecosystem. Despite having the largest *EUE* value, $LW_{out}$ is not a component of work since it is a passive response to the temperature state. Figure 4 displays the relationships of work versus temperature gradient between the atmosphere and land surface ($\Delta T/\Delta z$; Eq. 12) for the functional group scenarios at each site. Work performed within an ecosystem has a positive nonlinear relationship with temperature gradient across all scenarios. Further, each functional group scenario is fitted to a power function: $W=a(\Delta T/\Delta z)^b$. At each site a power law is observed; higher powers (*b*) correspond to functional groups with larger LAI, and except for the SRM-UN scenario, the highest power at each site corresponds to the multiple-functional-group scenario. This means that the work performed by the ecosystems with multiple functional groups has an exponentially greater response to marginal changes in temperature gradient as it increases.

To better understand the relationship between work and temperature gradients, we refer to the 1994 paper in which Schneider and Kay^10^ theoretically explored Silveston’s^36^ Bénard cell experiments of heating an enclosed fluid from below. They demonstrated that without self-organization, a system’s work performed from conduction alone has a linear relationship with the temperature gradient. However, when self-organization in the form of convection occurs at a critical point, Bénard cells form, the relationship becomes nonlinear, and more work is performed for each additional unit increase in gradient^10^. In this experiment, analysis of data in which no self-organization occurred at all demonstrated a linear relationship between work and gradient. Alternatively, when Bénard cells formed (i.e. self-organization occurred) power law relationships between work and gradient emerged with higher-degree powers corresponding to greater dissipation rates due to increased convection. We give this example not to study the emergence of the phenomena, but as a means of comparing the behavior of possible end states: with and without self-organization. If we take the functional group scenarios as possible end states with various levels of self-organization, the interpretation for each of our sites is consistent with those of the Bénard cells: with self-organization, there is a nonlinear relationship between work and gradient with larger exponents in the power law relationship corresponding to more advanced levels of self-organization. This yields exponentially more work performed in the more highly organized multiple-functional-group ecosystem scenarios. From the combined results of Schneider and Kay and Fig. 4, we infer that self-organization is the leading driver of the nonlinearity shown in work-gradient plots. This supports the proposition that the existence of multiple functional groups reflects a higher degree of self-organization that results in nonlinear increases of work performed in response to marginal increases in temperature gradient, reflecting thermodynamic advantage.

## Discussion

Through the concepts of entropy and work efficiency, this paper establishes a framework for identifying thermodynamic advantage for the spontaneous self-organization of ecosystems towards a vegetation structure that includes multiple functional groups. We identify decreased canopy temperature, increased LAI, and greater partitioning of energy towards $LW_{out}$ as important factors amplifying the entropy production of an ecosystem. From these factors, one can deduce the relative changes in entropy flux and, thus, changes in thermodynamic behavior of an ecosystem. Entropy provides insights into the total disorder of a system. The second law of thermodynamics requires that closed systems yield increases in entropy over time. Although, ecosystems are open thermodynamic systems, scientists have correlated greater entropy production as a driver of self-organization^11,37^. However, not all energy and hence entropy is productive in terms of thermodynamic advantage. Thus, we provide work efficiency as another important component of thermodynamic advantage and the directionality of self-organization.

Work efficiency captures the ability of an ecosystem to perform work based on the energy throughflow of the system. Work without context of incoming radiation doesn’t tell much about the performance of an ecosystem relative to others. Since all functional group scenarios at each site receive the same incoming radiation in this study, either work or work efficiency can be used as a metric of thermodynamic advantage. However, work efficiency is a more attractive metric for further study as it normalizes an ecosystem based on its local availability of energy, allowing for comparison of ecosystems across multiple climates. Thus, work efficiency provides promise for future research to compare ecosystems with varying energy availabilities and external environments directly.

Additionally, work efficiency measures an ecosystem’s ability to rapidly convert incoming radiation into alternate forms of energy that disperse throughout the ecosystem control volume and diminish the imposed temperature gradient. Work efficiency helps us understand the reorganization of available energy entering an ecosystem towards thermodynamically-productive uses—meaning depleting the imposed temperature gradient. According to thermodynamic theory, all systems work to decrease gradients of their state variables, which in turn drive the movement of energy and mass from high concentrations to low concentrations (i.e. high to low temperatures)^13^. In this study, work exhibits a nonlinear power law relationship with temperature gradient. This means that exponentially more work is performed to combat the greater temperature differences from the Earth surface to the atmosphere above the canopy. For the sites studied here, scenarios with multiple functional groups exhibit the highest power law, meaning that the MG structure is more efficient at depleting the driving temperature gradient. This is a demonstration of high work efficiency. Since structures that perform more work for a given temperature gradient have a thermodynamic advantage over those with lower efficiencies, ecosystems have a natural tendency to self-organize to this MG structure.

Because ecosystems are formed and evolve through a process of random fluctuations, there is a nonzero statistical probability for the existence of any possible vegetation structure or state. The state exhibiting thermodynamic advantage identifies the state with the highest probability of occurrence. This does not mean that the advantageous structure will always result. In all of our sites, the highest work efficiency corresponds to the existing MG scenario. Thus, the ecosystems exist in the highest probability thermodynamically-advantageous state.

The outcomes of this work provide valuable insight into the self-organization of natural ecosystems. Thus far, we have identified that when multiple functional groups coexist this structure exhibits a thermodynamic advantage over other possible individual functional group scenarios. Thus, ecosystems will have a higher probability of self-organizing towards this greater work efficiency state. Additionally, this work highlights new areas for further study. The framework of thermodynamic advantage through greater entropy production and work efficiency could be applied to other ecosystem structures, such as the existence of individual functional groups in nature. Further, this framework could help scientists understand how human-induced perturbations could impact thermodynamic behavior and alter the most advantageous state. Thus, we propose the concepts of entropy and work efficiency as valuable contributions to the basic understanding of the existence of a particular vegetation structure and present thermodynamic advantage as a tool for future use in understanding and studying the stability and behavior of ecosystem self-organization.

## Methods

### Experimental design

A multi-layer canopy-root-soil model (MLCan)^24,26,27^ is used to calculate the energy and entropy fluxes for three climatologically-different ecosystems containing multiple functional groups: water-limited Santa Rita Mesquite (SRM), energy-limited Willow Creek (WCR), and nutrient-limited Tapajos National Forest (TAP)^38^.

MLCan takes site-specific parameters and weather forcing data and computes the energy and entropy fluxes and temperatures for each of the ecosystem layers. Entropy calculations are based on both the energy fluxes and temperature of soil, air, and leaves (see *Entropy Calculations*). The model is run for a simulation period of 2 years (2004–2005) at a half-hourly timescale for SRM and WCR and an hourly timescale for TAP due to data availability. Weather forcing data was downloaded from FLUXNET2015: air temperature, air pressure, global radiation, precipitation, wind speed, friction velocity, and relative humidity^32–34^. Additional model input parameters can be found in Table S2 of the Supplementary Information.

The initial soil moisture and temperature profiles for each of the sites—and snow properties for WCR—were produced from a spin-up of the model. The WCR and TAP sites used 2004 LAI with 2003 forcing data for a spin-up of 2 years to provide the initial conditions for the beginning of the 2004 simulation. For the SRM site, the FLUXNET2015 data was not available for 2003, so 2004 data was used instead.

At each site, the model splits up the vegetation into plant functional groups. Domingues et al.^35^ demonstrates the importance of modeling ecosystems based on functional groups. For WCR and SRM, the vegetation is represented by understory herbaceous species and overstory trees. For TAP, a high biodiversity ecosystem in Amazonia, the vegetation is further divided and represented by four groups: understory tree, mid-canopy tree, upper-canopy tree, and upper-canopy liana^35^. See Table S1 of the Supplementary Information for functional group abbreviations.

The LAI data for all sites are taken from MODIS^39^ and calibrated based on site documentation (Fig. S4 of the Supplementary Information). The LAI is then partitioned into two or four components based on the number of functional groups at each site. Additional LAI information can be found in the Supplementary Information.

MLCan has been previously validated for each of the sites considered^30,40^. Since entropy cannot be directly measured, we provide a comparison of the model outputted latent heat fluxes with the observed fluxes at each site in Fig. S5 of the Supplementary Information for additional validation.

### Site descriptions

The SRM site is located on the Santa Rita Experimental Range in southern Arizona ($31.8214^{\circ }\hbox{N}$, $110.8661^{\circ }\hbox{W}$). SRM has a hot semi-arid climate and consists of woody savannas with mesquite trees (*Prosopis velutina* Woot.) and C4 grasses and subshrubs^40,41^.

The WCR (Willow Creek) site is located within the Chequamegon-Nicolet National Forest in northern Wisconsin ($45.8059^{\circ }\hbox{N}$, $90.0799^{\circ }\hbox{W}$) with a northern continental climate. It is a deciduous broadleaf forest dominated by sugar maple (*Acer saccharum* Marsh.) with understory shrubs, including bracken ferns (*Pteridium aquilinum*), and overstory seedlings and saplings^42–44^.

The TAP (Tapajos National Forest) site data is taken from the Santarem Km 67 Primary Forest site located in Belterra, Pará, Brazil ($2.8567^{\circ }\hbox{S}$, $54.9589^{\circ }\hbox{W}$). This evergreen broadleaf forest in Amazonian Brazil has a tropical monsoon climate with vegetation consisting of dozens of known tree species and lianas^30,35^.

### Entropy calculations

Entropy calculations are based on model-simulated temperature and energy at each of the 20 canopy layers and the soil-surface layer, and results are scaled up to the ecosystem level. No lateral exchange of fluxes are considered. The net sum of energy fluxes from all layers of the ecosystem is equivalent to the total flux of energy across the boundary of the control volume (Fig. S1 of the Supplementary Information). These energy fluxes include shortwave radiation (*SW*), longwave radiation (*LW*), latent heat (*LE*), and sensible heat (*H*). All results are categorized as the flux of energy at the boundary entering ($SW_{in}$, $LW_{in}$) or leaving ($SW_{out}$, $LW_{out}$, *LE*, *H*) the ecosystem. Because the total energy flux across the ecosystem boundary is equal to the sum across the canopy layers in the model, the total entropy flux across the boundary can also be taken as the cumulative sum of the entropy fluxes from all layers of the ecosystem.

Entropy flux calculations are summarized in Table 1. All energy variables have units of $\hbox{W/m}^2$, entropy variables are in $\hbox{W/m}^2\hbox{K}$, and temperatures are in K.

**Table 1:** Entropy calculations

Energy category	Symbol	Entropy equation
Latent heat	$J_{LE}$	$\frac{E_{LE}}{T_{eq,LE}}$
Sensible heat	$J_{H}$	$\frac{E_{H}}{T_{eq,H}}$
Direct shortwave	$J_{SW\,direct}$	$\frac{4}{3}\frac{E_{SW\,direct}}{T_{sun}}$
Incoming longwave	$J_{LW in}$	$\frac{4}{3}\frac{E_{LWin}}{T_{atm}} \times X(\epsilon )$
Outgoing longwave	$J_{LW out}$	$\frac{4}{3}\frac{E_{LWout}}{T_{eq,LWout}} \times X(\epsilon )$
Diffuse shortwave	$J_{SW diffuse}$	$\frac{4}{3}\frac{E_{SWdiffuse}}{T_{sun}} \times X(\xi )$

*X*: reflection factor as a function of $\xi$ or $\epsilon$ from Landsberg and Tongue^45^ and Wright et al.^46^.

$\xi$: dilution factor based on scattering^37,45,47,48^

$$\epsilon$$: emissivity

$T_{sun}$: temperature of the sun (5760 K)

$T_{atm}$: observed atmospheric temperature

$T_{eq,j}$: equivalent temperature of the system for energy category $j \in \{LW_{out}, LE, H\}$; see Eq. 5.

Entropy for *LE* and *H* calculations are based on simple heat transfer. The change in entropy is:

1 $$\begin{aligned} dS=\frac{dQ}{T} \end{aligned}$$

where *dQ* is change in heat and *T* is temperature^49^. Thus, the flux of entropy for a given energy flux (*E*) across a boundary is:

2 $$\begin{aligned} J=\frac{E}{T} . \end{aligned}$$

However, thermal radiation (*SW* and *LW*) cannot be treated this simply. The entropy flux for blackbody radiation is:

3 $$\begin{aligned} J_{BR} = \frac{4}{3} \sigma T^3 = \frac{4}{3} \frac{E_{BR}}{T} \end{aligned}$$

where $\sigma$ is the Stefan–Boltzmann constant, and $E_{BR}$ is the blackbody radiation flux defined as $\sigma T^4$ from the Stefan–Boltzmann Law^48,49^.

*SW* is considered blackbody radiation, and entropy fluxes for direct shortwave radiation ($J_{SW\,direct}$) can be obtained by Eq. 3. However, *LW* is considered non-blackbody radiation, also called ‘diluted blackbody radiation’, which must include an additional factor $X(\epsilon )$ to account for the entropy produced during the ‘diluted emission’ of radiation given by an object’s emissivity, $\epsilon$. This factor is defined as^45,46^:

4 $$\begin{aligned} X(\epsilon ) = 1-\Big [\frac{45}{4\pi ^4}\ln {(\epsilon )}(2.336-0.26\epsilon )\Big ]. \end{aligned}$$

Although $SW_{diffuse}$ is still a blackbody radiation, it has been demonstrated^47^ that the entropy flux due to $SW_{diffuse}$ can be treated similarly to non-blackbody radiation with a new variable, $\xi$, in place of emissivity. $\xi$ is the ‘dilution factor’ of radiation due to scattering, meaning it is the ratio of diffuse solar radiance on Earth’s surface to solar radiance in extraterrestrial space^47^. Since diluted blackbody radiation ($SW_{diffuse}$) is mathematically equivalent to non-blackbody radiation (*LW*) when the dilution factor is equal to the emissivity, $\xi$ can also be plugged into Eq. 4 to solve for the amplifying factor of entropy production due to scattering, $X(\xi )$^37,45,46,48^.

Each of the entropy calculations in Table 1 have a temperature value corresponding to the temperature of the energy’s source. For instance, shortwave radiation originates from the sun, so the source temperature in its entropy equations is $T_{sun}$. Likewise, longwave radiation is assumed to originate from the atmosphere, leading to a corresponding temperature of $T_{atm}$. However, $LW_{out}$, *LE*, and *H* do not have a single source location, so we must calculate an equivalent temperature ($T_{eq}$) for each energy category based on the modeled temperatures and weighted contribution of each layer to the total energy flux at the ecosystem boundary. The equivalent temperatures for these three energy categories are calculated as follows:

5 $$\begin{aligned} T_{eq,j} = \sum _{k=1}^{21}[T_{k} \times \omega _{j, k}] \end{aligned}$$

where $T_{eq,j}$ is the equivalent temperature of energy category *j* such that $j \in \{LW_{out}, LE, H\}$. *k* refers to the layer in the ecosystem such that layers 1-20 are the canopy layers, and layer 21 refers to the ground surface. $T_k$ is the temperature of layer *k*, and $\omega _{j,k}$ is the weight of energy category *j* coming from layer *k* given by:

6 $$\begin{aligned} \omega _{j,k} = \frac{E_{j,k}}{E_{j,eco}} \end{aligned}$$

where $E_{j,k}$ is the energy *j* leaving layer k, and $E_{j,eco}$ is the total energy *j* leaving the ecosystem.

The total entropy flux of the ecosystem ($J_{eco}$) is calculated by summing the energy categories:

7 $$\begin{aligned} J_{eco} = \sum J_j + J_{SWout} \end{aligned}$$

where $J_{SWout}$ is the entropy flux of diffuse shortwave radiation leaving the ecosystem. The entropy flux per unit energy (*EUE*) is another way to view the thermodynamic state of ecosystem vegetation. *EUE* is calculated as:

8 $$\begin{aligned} EUE_{j} = \frac{J_{j}}{E_{j}} \end{aligned}$$

where $EUE_{j}$ is the entropy per unit energy in 1/K of energy category *j*. It follows that the corresponding $EUE_{SWout} = J_{SWout}/E_{SWout}$, and the total ecosystem *EUE* is:

9 $$\begin{aligned} EUE_{eco} = \frac{\sum J_j + J_{SWout}}{\sum E_j + E_{SWout}}. \end{aligned}$$

### Work calculations

Work in an ecosystem represents the energy required to directly perform motion in the form of heat, effectively decreasing the temperature gradient within the ecosystem. We assume that *LE* and *H* are the primary regulators of temperature within a natural ecosystem, and $LW_{out}$ is wasted energy. Additionally, we assume that the bottom of the control volume is sufficiently deep such that the temperature at the boundary is consistent and there is no loss of heat (i.e. ground heat flux is ignored). Thus, work is estimated and calculated directly from *LE*, *H*, and change in internal energy due to photosynthesis, $\Delta Q$:

10 $$\begin{aligned} W = LE+H+\Delta Q \end{aligned}$$

where $\Delta Q$ is significantly less than *LE* and *H* and can be ignored. So work can be simplified to:

11 $$\begin{aligned} W = LE+H. \end{aligned}$$

Since work represents the ability of an ecosystem’s vegetation to deplete the driving temperature gradient imposed upon the ecosystem, our analysis compares work with temperature gradient. We define temperature gradient as:

12 $$\begin{aligned} \frac{\Delta T}{\Delta z} = \frac{T_{surf}-T_{air}}{h_e} \end{aligned}$$

where $T_{surf}$ is the temperature of the soil surface, $T_{air}$ is the temperature of the air in the top layer of the ecosystem, and $h_e$ is the ecosystem height (see Table S2 in the Supplementary Information).

Work efficiency is the work performed for the amount of radiation entering the ecosystem defined as:

13 $$\begin{aligned} WE = \frac{LE+H}{E_{SWin} + E_{LWin}} = \frac{W}{E_{in}}. \end{aligned}$$

Since each vegetation functional group partitions energy differently among the energy categories, work efficiency is a good way to compare thermodynamic behavior across model scenarios at each site in a normalized way.

### Statistical analysis

To determine if the differences of entropy flux and work efficiency among scenarios at each site are statistically significant, we perform two separate tests for entropy flux and work efficiency. Since entropy flux distributions are positively skewed (Fig. 1a), we use the variance as an indicator of the difference between them. To this end we use the distribution-free Miller Jackknife (MJ) significance test^50,51^ for variance that does not assume that the distributions come from populations with the same median. However, the work efficiency distributions exhibit no such pattern (Fig. 1b), and, therefore, we use the two-sample Kolmogorov–Smirnov (KS) test, which measures the maximum absolute difference between two empirical cumulative distribution functions (CDF)^52–54^.

First, the entropy flux variances are compared with the MJ test. Because functional group scenarios at each site are bounded on the lower end by similar values, if a distribution has a larger variance than another, then the two populations cannot be considered as coming from the same continuous distribution, and the distribution with a larger variance generally consists of larger values. For each site we test the null hypothesis, $H_0$, that the distribution of multiple-functional-group entropy fluxes and the distribution for each of its single-functional-groups have the same variance. This is done with each functional group present at each site (Table S1). The alternate hypothesis, $H_{A1}$, states that the distribution of entropy fluxes from the multiple-functional-group has a larger variance than that of the corresponding single-functional-group, meaning that the two populations do not belong to the same distribution and the multi-group scenario consists of larger values than the single-group scenario. The results from this test, shown in Table 2, indicate that $H_0$ is rejected in favor of $H_{A1}$ at the 5% level ($p<0.05$) for all scenarios except for the WCR-OT scenario. This indicates that for these ecosystems the distributions of entropy fluxes consist of larger values when multiple functional groups are present.

**Table 2:** Significance tests

Site	Funct. group	$H_{A1}$		$H_{A2}$		$H_{A3}$		$H_{A4}$	
		*p* value	Reject $H_0$?	*p* value	Reject $H_0$?	*p* value	Reject $H_0$?	*p* value	Reject $H_0$?
SRM	UN	0.004	Yes			$\sim$ 0	Yes		
	OT	0.017	Yes			0.002	Yes		
WCR	UN	$\sim$ 0	Yes			$\sim$ 0	Yes		
	OT	0.606	No	0.394	No	0.934	No	0.915	No
TAP	UN	$\sim$ 0	Yes			$\sim$ 0	Yes		
	MT	$\sim$ 0	Yes			$\sim$ 0	Yes		
	OT	$\sim$ 0	Yes			$\sim$ 0	Yes		
	L	$\sim$ 0	Yes			$\sim$ 0	Yes		

$H_0$ is rejected if $p<0.05$ at the 5% significance level.

*Entropy—Miller Jackknife test of variance*

$H_{A1}$: The entropy flux results from multiple functional groups have a variance **larger than** the single-functional-group.

$H_{A2}$: The entropy flux results from multiple functional groups have a variance **smaller than** the single-functional-group.

*Work efficiency—two-sample Kolmogorov–Smirnov test*

$H_{A3}$: The work efficiency results from multiple functional groups have a CDF **smaller than** the single-functional-group (i.e. values are generally larger).

$H_{A4}$: The work efficiency results from multiple functional groups have a CDF **larger than** the single-functional-group (i.e. values are generally smaller).

Using the KS test to compare work efficiency distributions for each site, we test the null hypothesis, $H_0$, that the multiple-functional-group measures of work efficiency and those for each of its single-functional-groups are from the same continuous distribution, or population. The alternate hypothesis, $H_{A3}$, states that the CDFs of the entropy flux from the multi-group scenario are smaller than those from the corresponding single-groups, meaning that the multi-group scenarios consist of values that are larger than their associated single-group scenarios. The results from this test, shown in Table 2, indicate that $H_0$ is rejected in favor of $H_{A3}$ at the 5% level ($p<0.05$) for all scenarios except for the WCR-OT scenario. This indicates that the distributions for work efficiency are indeed larger when multiple functional groups are present in these ecosystems, as indicated by a smaller CDF (Fig. S3 of the Supplementary Information).

However, the tests of comparison for the WCR-OT scenario for both work efficiency and entropy flux distributions have *p* values larger than 0.05 (i.e. $H_0$ cannot be rejected at the 5% level). This means that we cannot say that the WCR multi-group entropy flux distribution has a variance larger than the OT single-group distribution or the multi-group work efficiency scenario comes from a larger distribution than the OT single-group scenario. This is not entirely surprising, as there is very little difference in LAI between these two scenarios; the maximum difference in LAI is about 0.2 (Fig. S4 in the Supplementary Information), only 3% compared to the total WCR-MG LAI. This small increase in LAI from the single to the multi-group scenario provides less opportunity for increased energy dissipation and hence entropy production due to the smaller understory. Thus for completeness, we also perform the MJ and KS tests in the opposite direction for the WCR-OT scenario with the following alternative hypotheses. For the MJ test on entropy flux variances, $H_{A2}$ states that the distribution of entropy fluxes from the WCR multiple-functional-group has a smaller variance than that of the OT single-functional-group, meaning that the two populations do not belong to the same distribution and the multi-group scenario consists of smaller values than the single-group scenario. For the KS test on work efficiency, $H_{A4}$ states that the CDF of the entropy fluxes from the WCR multiple-functional-group is larger than the CDF from the OT single-functional-group, meaning that the multi-group scenario consists of values that are smaller than the single-group scenario. The results from both tests, shown in Table 2, indicate that we again cannot reject $H_0$ at the 5% significance level for WCR-OT. Thus, although the WCR multi-group scenario compared to the OT scenario does not have a significantly greater entropy flux variance or a greater work efficiency distribution, it also does not have less variance or a smaller distribution. Overall, the test results indicate that multiple-functional-groups have either greater or similar values of entropy flux and work efficiency than the modeled scenarios of their individual functional groups.

## Supplementary information

Supplementary Information.

## References

[1] Prigogine I, Wiame J-M (1946). Biologie et thermodynamique des phénomènes irréversibles. Experientia.

[2] Prigogine, I. *Étude thermodynamique des phénomènes irréversibles* (Ph.D. Brussels University, 1947).

[3] Von Bertalanffy L, Woodger JH (1938). Modern Theories of Development.

[4] Von Bertalanffy L (1950). The theory of open systems in physics and biology. Science.

[5] Prigogine I (1961). Introduction to Thermodynamics of Irreversible Processes.

[6] Schneider, E. D. & Kay, J. J. Order from disorder: the thermodynamics of complexity in biology. In *What is Life? The Next Fifty Years: Speculations on the Future of Biology* 161–172 (1995). 10.1017/CBO9780511623295.013.

[7] Olson JS (1963). Energy storage and the balance of producers and decomposers in ecological systems. Ecology.

[8] Holling CS (1973). Resilience and stability of ecological systems. Annu. Rev. Ecol. Syst..

[9] Paltridge GW (1979). Climate and thermodynamic systems of maximum dissipation. Nature.

[10] Schneider ED, Kay JJ (1994). Life as a manifestation of the second law of thermodynamics. Math. Comput. Model..

[11] Kleidon A (2016). Thermodynamic Foundations of the Earth System.

[12] Schneider ED, Kay JJ (1994). Complexity and thermodynamics: towards a new ecology. Futures.

[13] Kondepudi D, Prigogine I (2014). Modern Thermodynamics: From Heat Engines to Dissipative Structures.

[14] Odum EP (1968). Energy flow in ecosystems: a historical review. Am. Zool..

[15] Odum EP (1976). Energy, ecosystem development and environmental risk. J. Risk Insur..

[16] Johnson, L. The thermodynamics of ecosystems. In *The Natural Environment and the Biogeochemical Cycles*, 1–47 (Springer, 1990).

[17] Jørgensen SE, Nielsen SN, Mejer H (1995). Emergy, environ, exergy and ecological modelling. Ecol. Model..

[18] Bendoricchio G, Jørgensen SE (1997). Exergy as goal function of ecosystems dynamic. Ecol. Model..

[19] Svirezhev YM (2000). Thermodynamics and ecology. Ecol. Model..

[20] Jørgensen SE, Fath BD (2004). Application of thermodynamic principles in ecology. Ecol. Complex..

[21] Jorgensen SE, Svirezhev YM (2004). Towards a Thermodynamic Theory for Ecological Systems.

[22] Jørgensen SE (2006). Eco-exergy as Sustainability.

[23] Lavorel S, McIntyre S, Landsberg J, Forbes T (1997). Plant functional classifications: from general groups to specific groups based on response to disturbance. Trends Ecol. Evol..

[24] Drewry DT (2010). Ecohydrological responses of dense canopies to environmental variability: 1. Interplay between vertical structure and photosynthetic pathway. J. Geophys. Res. Biogeosci..

[25] Drewry DT (2010). Ecohydrological responses of dense canopies to environmental variability: 2. Role of acclimation under elevated CO2. J. Geophys. Res. Biogeosci..

[26] Le PVV, Kumar P, Drewry DT (2011). Implications for the hydrologic cycle under climate change due to the expansion of bioenergy crops in the Midwestern United States. Proc. Natl. Acad. Sci..

[27] Le PVV, Kumar P, Drewry DT, Quijano JC (2012). A graphical user interface for numerical modeling of acclimation responses of vegetation to climate change. Comput. Geosci..

[28] Quijano JC, Kumar P, Drewry DT, Goldstein A, Misson L (2012). Competitive and mutualistic dependencies in multispecies vegetation dynamics enabled by hydraulic redistribution. Water Resour. Res..

[29] Quijano JC, Kumar P, Drewry DT (2013). Passive regulation of soil biogeochemical cycling by root water transport. Water Resour. Res..

[30] Quijano JC, Kumar P (2015). Numerical simulations of hydraulic redistribution across climates: the role of the root hydraulic conductivities. Water Resour. Res..

[31] Pastorello G (2020). The FLUXNET2015 dataset and the ONEFlux processing pipeline for eddy covariance data. Sci. Data.

[32] Scott, R. FLUXNET2015 US-SRM Santa Rita Mesquite (2004–2014). 10.18140/FLX/1440090.

[33] Desai, A. FLUXNET2015 US-WCr Willow Creek (1999–2014). 10.18140/FLX/1440095.

[34] Saleska, S. FLUXNET2015 BR-Sa1 Santarem-Km67-Primary Forest (2002–2011). 10.18140/FLX/1440032.

[35] Domingues TF, Martinelli LA, Ehleringer JR (2007). Ecophysiological traits of plant functional groups in forest and pasture ecosystems from eastern Amazonia, Brazil. Plant Ecol..

[36] Silveston, P. Warmedurchange in horizontalen flassigkeitschichtem. *Heat Changes in Horizontal Silicon Oil*. PhD thesis, Techn. Hochsch. Muenchen, Germany (1957).

[37] Quijano, J. C. *Coupled Dynamics of Above- and Below-ground Interactions in the Critical Zone* (Ph.D. University of Illinois at Urbana-Champaign, 2013).

[38] McGroddy ME, Silver WL, de Oliveira RC (2004). The effect of phosphorus availability on decomposition dynamics in a seasonal lowland Amazonian forest. Ecosystems.

[39] ORNL DAAC 2018. MODIS and VIIRS Land Products Global Subsetting and Visualization Tool. ORNL DAAC, Oak Ridge, Tennessee, USA. Accessed April, 2019. Subset obtained for MOD15A2H product at −3.01803, −54.9714400, time period: 2000 to 2018, and subset size: 0.5 × 0.5 km (2018). 10.3334/ORNLDAAC/1379.

[40] Lee E (2018). Impact of hydraulic redistribution on multispecies vegetation water use in a semiarid savanna ecosystem: An experimental and modeling synthesis. Water Resour. Res..

[41] Scott RL, Jenerette GD, Potts DL, Huxman TE (2009). Effects of seasonal drought on net carbon dioxide exchange from a woody-plant-encroached semiarid grassland. J. Geophys. Res. Biogeosci..

[42] Curtis PS (2002). Biometric and eddy-covariance based estimates of annual carbon storage in five eastern North American deciduous forests. Agric. For. Meteorol..

[43] Cook BD (2004). Carbon exchange and venting anomalies in an upland deciduous forest in northern Wisconsin, USA. Agric. For. Meteorol..

[44] Ewers B, Mackay D, Tang J, Bolstad P, Samanta S (2008). Intercomparison of sugar maple (Acer saccharum Marsh.) stand transpiration responses to environmental conditions from the Western Great Lakes Region of the United States. Agric. For. Meteorol..

[45] Landsberg P, Tonge G (1979). Thermodynamics of the conversion of diluted radiation. J. Phys. A Math. Gen..

[46] Wright S, Scott D, Haddow J, Rosen M (2001). On the entropy of radiative heat transfer in engineering thermodynamics. Int. J. Eng. Sci..

[47] Aoki I (1982). Radiation entropies in diffuse reflection and scattering and application to solar radiation. J. Phys. Soc. Jpn..

[48] Wu W, Liu Y (2010). Radiation entropy flux and entropy production of the Earth system. Rev. Geophys..

[49] Planck M (2013). The Theory of Heat Radiation.

[50] Hollander MA, Wolfe D, Chicken E (2015). The two-sample dispersion problem and other two-sample problems. Nonparametric Stat. Methods.

[51] Miller RG (1968). Jackknifing variances. Ann. Math. Stat..

[52] Darling DA (1957). The Kolmogorov–Smirnov, Cramer–von Mises Tests. Ann. Math. Stat..

[53] Young IT (1977). Proof without prejudice: use of the Kolmogorov–Smirnov test for the analysis of histograms from flow systems and other sources. J. Histochem. Cytochem..

[54] Gibbons J, Chakraborti S (2010). Nonparametric Statistical Inference.

