# Supplementary material for: Discerning the thermodynamic feasibility of the spontaneous coexistence of multiple functional vegetation groups
Source: Sci Rep. 2020 Oct 27;10:18321. doi: 10.1038/s41598-020-75050-4 (PMC7591582; doi:10.1038/s41598-020-75050-4)
Supplement: Supplementary file 1 — Supplementary Information. [file 41598_2020_75050_MOESM1_ESM.pdf]

Discerning the thermodynamic feasibility of the spontaneous coexistence of multiple functional vegetation groups

Meredith Richardson and Praveen Kumar

Supplementary Materials

Supplementary Text
Figs. S1 to S5
Tables S1 to S2

Supplementary Text

Figure S1 provides a conceptual diagram of the ecosystem control volume and fluxes modeled in this study. Figure S2 shows the locations overlaid upon the mean annual precipitation of the study sites considered. Table S1 outlines the abbreviations for the vegetation considered within each of the functional groups for each site.

Further Results

The Two-sample Kolmogorov-Smirnov test measures the maximum absolute vertical distance between two cumulative distribution functions (CDF)[1–3]. Figure S3 displays the work efficiency CDF's for all functional group scenarios at each site. This figure demonstrates that the multiple-functional-group (MG) scenario at each site has the largest values (aside from WCR-OT; see Table 1 in the main text) due to the smaller CDF's indicated by a significantly large vertical distance between MG and the other functional groups.

Additional Parameters and Calculations

All variables except friction velocity and relative humidity were already gap-filled in the available Fluxnet2015 dataset[4–6]. In order to fill gaps in the relative humidity, variables were run through REddyProc online tool[7]. The interpolation scheme fills gaps based on other variables available at the same timestep. The vapor pressure deficit was then calculated from relative humidity (RH) and air temperature (T_a) at each timestep.

Additional input parameters for MLCan are displayed in Table S2.

The leaf area index (LAI) data for all sites are taken from MODIS[8] and calibrated and partitioned based on site documentation (Fig. S4). The LAI for each site is also interpolated for the appropriate timescale based on data fitted to one or composite polynomial functions depending on the shape. Before fitting, outliers past two standard deviations were removed.

The LAI for Santa Rita mesquite (SRM) was calibrated to the site and partitioned based on Lee et al.[9] for two years.

LAI for Willow Creek (WCR) is taken from the MODIS network and compared with field measurements. The understory LAI was taken from a local shrubland near the WCR site, given as 0.2[10,11]. This site, also located in the Chequamegon-Nicolett National Forest, has been frequently used to compare vegetation responses with WCR and several other local sites in the forest[10,11]. This LAI of 0.2 is assumed as the maximum understory value for the year, and the overstory LAI was extrapolated from the difference in the interpolated total LAI curve from MODIS and this understory LAI value. Field measurements for total LAI for 2000 to 2006 in WCR ranges from 0.0 to 5.3[12,13], similar to the MODIS range of 0.1 to 5.8. Thus, the curves were fitted to the original MODIS data. The LAI curve for WCR was created from a composite of five different polynomial functions. The winters (low LAI) are characterized by second degree curves, and the summers (high LAI) are characterized by fourth degree curves.

LAI for Tapajos National Forest (TAP) is obtained from MODIS data and compared with and partitioned based on Domingues et al.[14]. Quality issues with MODIS pixels existed over this site due to the denser cloud cover in the wet season. Shabanov et al.[15] and Myneni et al.[16] compare field measurements with MODIS algorithm performance; the range of values observed at these field sites was 5.4 to 7.0. Alternatively, field studies by Joetzjer et al.[17] and Brando et al.[18] indicate that the range of acceptable LAI for 2004 is from 4.7 to 5.7 and up to around 6.3 for 2005. The resulting LAI we use for TAP is based on an interpolated scheme for the MODIS data scaled to the range of values that satisfy a compilation of the field studies (4.7-6.3). A curve was fitted to the original MODIS

data and scaled up to fit within this published range. The resulting curve is a fifth-order polynomial function over the entire two year period.

Leaf area density (LAD) (i.e. the normalized vertical distribution of LAI) was solved for each functional group by different techniques based on data availability. LAD for WCR was taken from Radtke and Bolstad[19], and Weibull distributions were fitted to this data for each functional group[20,21]. The same process was repeated for TAP, where the LAD was taken from Stark et al.[22]. Understory was assumed to be 10% of the total LAD distribution up to its maximum height, 2m. Lianas take up a smaller distribution of the LAI than overstory and mid-canopy trees[23]; thus, the remaining LAD distribution was then partitioned at each layer 2-parts MT, 2-parts OT, and 1-part up to the mean maximum heights of each functional group described in Domingues et al.[14]. LAD for SRM was taken from Lee et al.[9].

MLCan has been previously validated for each of these sites[9,24]. For the present study, model validation for latent heat is shown in Fig. S5.

References

1. Darling, D. A. The Kolmogorov-Smirnov, Cramer-von Mises Tests. *The Annals Math. Stat.* **28**, 823–838, DOI: 10.2307/2237048 (1957).
2. Young, I. T. Proof without prejudice: use of the Kolmogorov-Smirnov test for the analysis of histograms from flow systems and other sources. *J. Histochem. & Cytochem.* **25**, 935–941, DOI: 10.1177/25.7.894009 (1977).
3. Gibbons, J. & Chakraborti, S. *Nonparametric Statistical Inference, Fifth Edition* (Taylor & Francis, 2010).
4. Desai, A. FLUXNET2015 US-WCr Willow Creek., DOI: 10.18140/FLX/1440095 (1999-2014).
5. Saleska, S. FLUXNET2015 BR-Sa1 Santarem-Km67-Primary Forest., DOI: 10.18140/FLX/1440032 (2002-2011).
6. Scott, R. FLUXNET2015 US-SRM Santa Rita Mesquite., DOI: 10.18140/FLX/1440090 (2004-2014).
7. Wutzler, T. *et al.* Basic and extensible post-processing of eddy covariance flux data with reddyproc. *Biogeosciences Discuss.* **15**, 5015–5030, DOI: 10.5194/bg-15-5015-2018 (2018).
8. ORNL DAAC 2018. MODIS and VIIRS Land Products Global Subsetting and Visualization Tool. ORNL DAAC, Oak Ridge, Tennessee, USA. Accessed April, 2019. Subset obtained for MOD15A2H product at -3.01803, -54.9714400, time period: 2000 to 2018, and subset size: 0.5 x 0.5 km, DOI: 10.3334/ORNLDAAC/1379 (2018).
9. Lee, E. *et al.* Impact of hydraulic redistribution on multispecies vegetation water use in a semiarid savanna ecosystem: An experimental and modeling synthesis. *Water Resour. Res.* **54**, 4009–4027, DOI: 10.1029/2017WR021006 (2018).
10. Desai, A. R. *et al.* Influence of vegetation and seasonal forcing on carbon dioxide fluxes across the Upper Midwest, USA: Implications for regional scaling. *agricultural forest meteorology* **148**, 288–308, DOI: 10.1016/j.agrformet.2007.08.001 (2008).
11. Noormets, A. *et al.* Moisture sensitivity of ecosystem respiration: comparison of 14 forest ecosystems in the Upper Great Lakes Region, USA. *Agric. For. Meteorol.* **148**, 216–230, DOI: 10.1016/j.agrformet.2007.08.002 (2008).
12. Cook, B. D. *et al.* Carbon exchange and venting anomalies in an upland deciduous forest in northern Wisconsin, USA. *Agric. For. Meteorol.* **126**, 271–295, DOI: 10.1016/j.agrformet.2004.06.008 (2004).
13. Desai, A. R. Climatic and phenological controls on coherent regional interannual variability of carbon dioxide flux in a heterogeneous landscape. *J. Geophys. Res. Biogeosciences* **115**, DOI: 10.1029/2010JG001423 (2010).
14. Domingues, T. F., Martinelli, L. A. & Ehleringer, J. R. Ecophysiological traits of plant functional groups in forest and pasture ecosystems from eastern Amazonia, Brazil. *Plant Ecol.* **193**, 101–112, DOI: 10.1007/s11258-006-9251-z (2007).

15. Shabanov, N. V. *et al.* Analysis and optimization of the modis leaf area index algorithm retrievals over broadleaf forests. *IEEE Transactions on Geosci. Remote. Sens.* **43**, 1855–1865, DOI: 10.1109/TGRS.2005.852477 (2005).

16. Myneni, R. B. *et al.* Large seasonal swings in leaf area of Amazon rainforests. *Proc. Natl. Acad. Sci.* **104**, 4820–4823, DOI: 10.1073/pnas.0611338104 (2007).

17. Joetzjer, E. *et al.* Predicting the response of the amazon rainforest to persistent drought conditions under current and future climates: a major challenge for global land surface models. *Geosci. Model. Dev.* **7**, 2933–2950, DOI: 10.5194/gmd-7-2933-2014 (2014).

18. Brando, P. M. *et al.* Drought effects on litterfall, wood production and belowground carbon cycling in an Amazon forest: results of a throughfall reduction experiment. *Philos. Transactions Royal Soc. Lond. B: Biol. Sci.* **363**, 1839–1848, DOI: 10.1098/rstb.2007.0031 (2008).

19. Radtke, P. J. & Bolstad, P. V. Laser point-quadrat sampling for estimating foliage-height profiles in broad-leaved forests. *Can. J. For. Res.* **31**, 410–418, DOI: 10.1139/x00-182 (2001).

20. Weibull, W. A statistical distribution function of wide applicability. *J. applied mechanics* **103**, 293–297 (1951).

21. Bartkute, V. & Sakalauskas, L. The method of three-parameter weibull distribution estimation. *Acta et commentationes Univ. Tartuensis de mathematica* **12**, 65–78 (2008).

22. Stark, S. C. *et al.* Amazon forest carbon dynamics predicted by profiles of canopy leaf area and light environment. *Ecol. Lett.* **15**, 1406–1414, DOI: 10.1111/j.1461-0248.2012.01864.x (2012).

23. Rodríguez-Ronderos, M. E., Bohrer, G., Sanchez-Azofeifa, A., Powers, J. S. & Schnitzer, S. A. Contribution of lianas to plant area index and canopy structure in a Panamanian forest. *Ecology* **97**, 3271–3277, DOI: 10.1002/ecy.1597 (2016).

24. Quijano, J. C. & Kumar, P. Numerical simulations of hydraulic redistribution across climates: The role of the root hydraulic conductivities. *Water Resour. Res.* **51**, 8529–8550, DOI: 10.1002/2014WR016509 (2015).

25. Hijmans, R. J., Cameron, S. E., Parra, J. L., Jones, P. G. & Jarvis, A. Very high resolution interpolated climate surfaces for global land areas. *Int. J. Climatol. A J. Royal Meteorol. Soc.* **25**, 1965–1978, DOI: 10.1002/joc.1276 (2005).

26. Nishida, K. & Hanba, Y. T. Photosynthetic response of four fern species from different habitats to drought stress: relationship between morpho-anatomical and physiological traits. *Photosynthetica* **55**, 689–697, DOI: 10.1007/s11099-017-0694-3 (2017).

27. Raczka, B., Dietze, M. C., Serbin, S. P. & Davis, K. J. What limits predictive certainty of long-term carbon uptake? *J. Geophys. Res. Biogeosciences* **123**, 3570–3588, DOI: 10.1029/2018JG004504 (2018).

28. Kubiske, M. E., Zak, D. R., Pregitzer, K. S. & Takeuchi, Y. Photosynthetic acclimation of overstory Populus tremuloides and understory Acer saccharum to elevated atmospheric CO2 concentration: interactions with shade and soil nitrogen. *Tree Physiol.* **22**, 321–329, DOI: 10.1093/treephys/22.5.321 (2002).

29. Bolstad, P. V., Davis, K. J., Martin, J., Cook, B. & Wang, W. Component and whole-system respiration fluxes in northern deciduous forests. *Tree physiology* **24**, 493–504, DOI: 10.1093/treephys/24.5.493 (2004).

30. Saito, M., Maksyutov, S., Hirata, R. & Richardson, A. D. An empirical model simulating diurnal and seasonal CO2 flux for diverse vegetation types and climate conditions. *Biogeosciences* **6**, 585–599, DOI: 10.5194/bg-6-585-2009 (2009).

31. Melton, J. R., Shrestha, R. K. & Arora, V. K. The influence of soils on heterotrophic respiration exerts a strong control on net ecosystem productivity in seasonally dry Amazonian forests. *Biogeosciences* **12**, 1151–1168, DOI: 10.5194/bg-12-1151-2015 (2015).

32. Schenk, H. J. & Jackson, R. B. The global biogeography of roots. *Ecol. monographs* **72**, 311–328, DOI: 10.1890/0012-9615(2002)072[0311:TGBOR]2.0.CO;2 (2002).

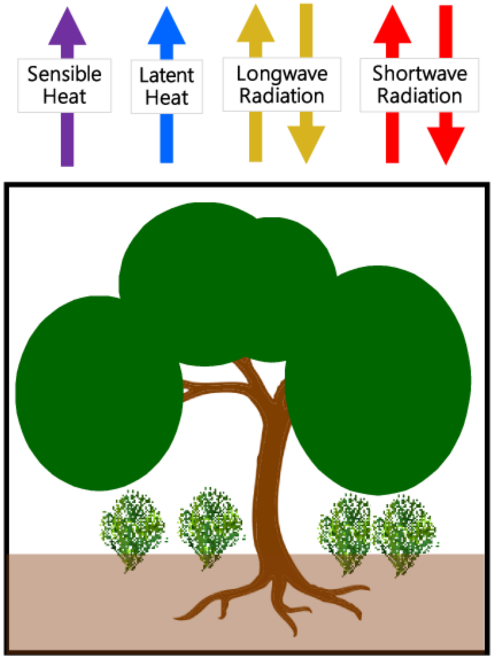

Figure S1. Schematic diagram of ecosystem control volume and energy fluxes considered for a situation with two functional groups. The lower boundary corresponds to a constant temperature with zero thermal gradient.

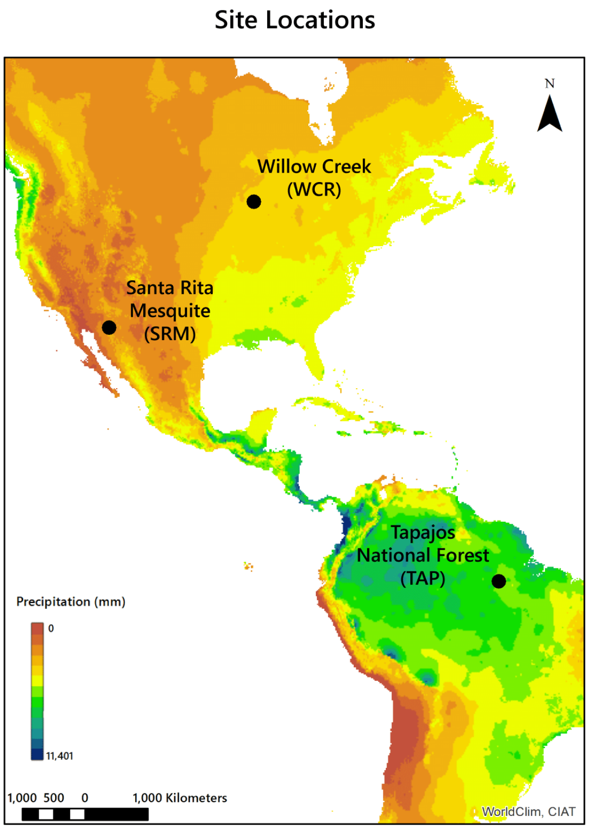

Figure S2. Map of site locations existing in Wisconsin and Arizona, United States, and Pará, Brazil. Background map displaying mean annual precipitation was adapted from Hijmans et al.[25].

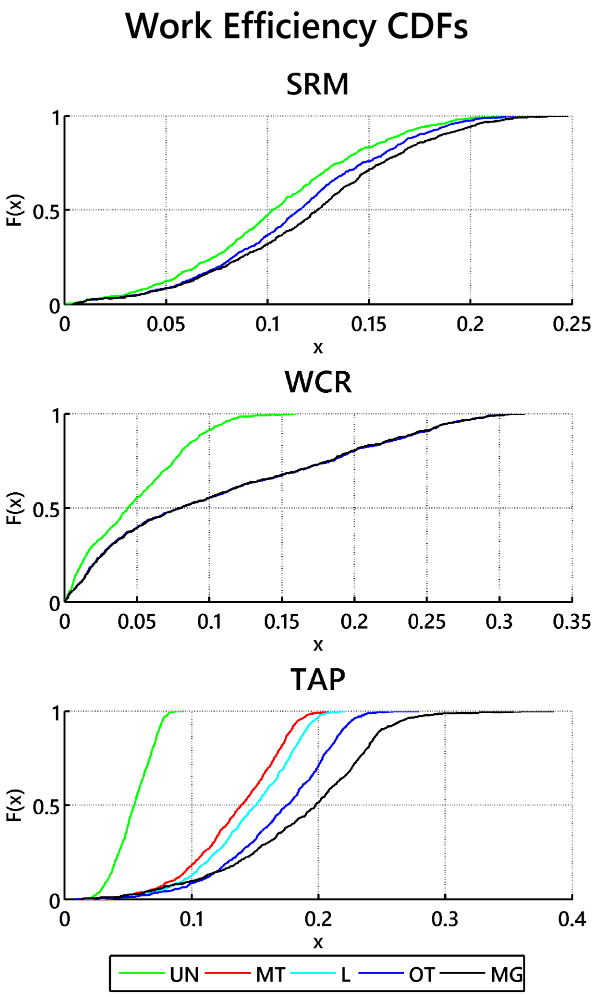

Figure S3. The cumulative distribution functions of work efficiency associated with each functional group and coexisting multi-functional vegetation groups (see Table 1 in the main text).

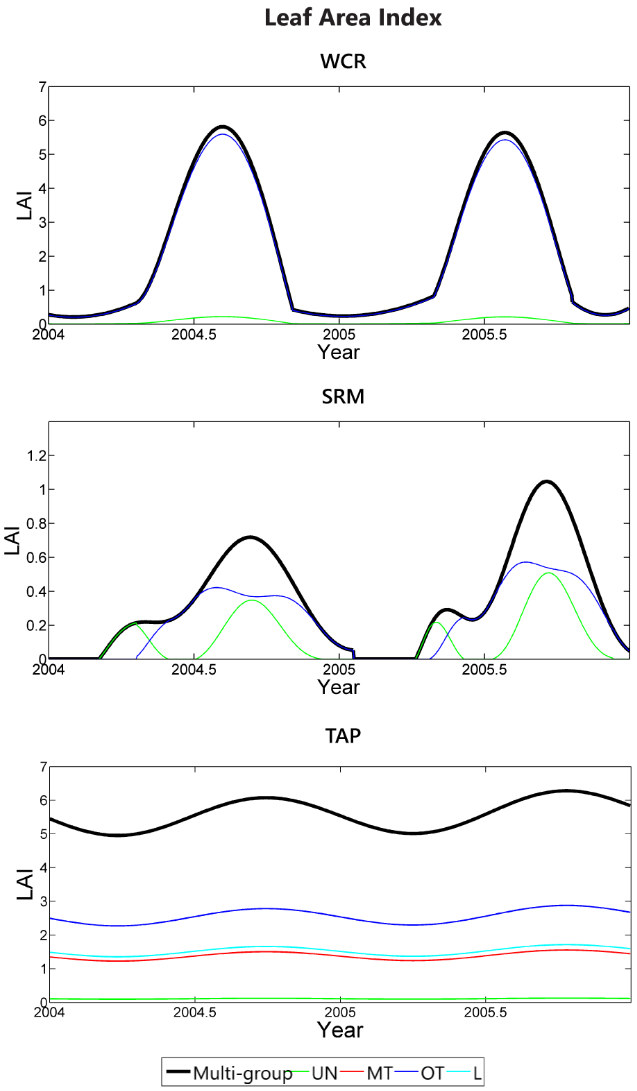

Figure S4. Leaf area index (LAI) for all sites.

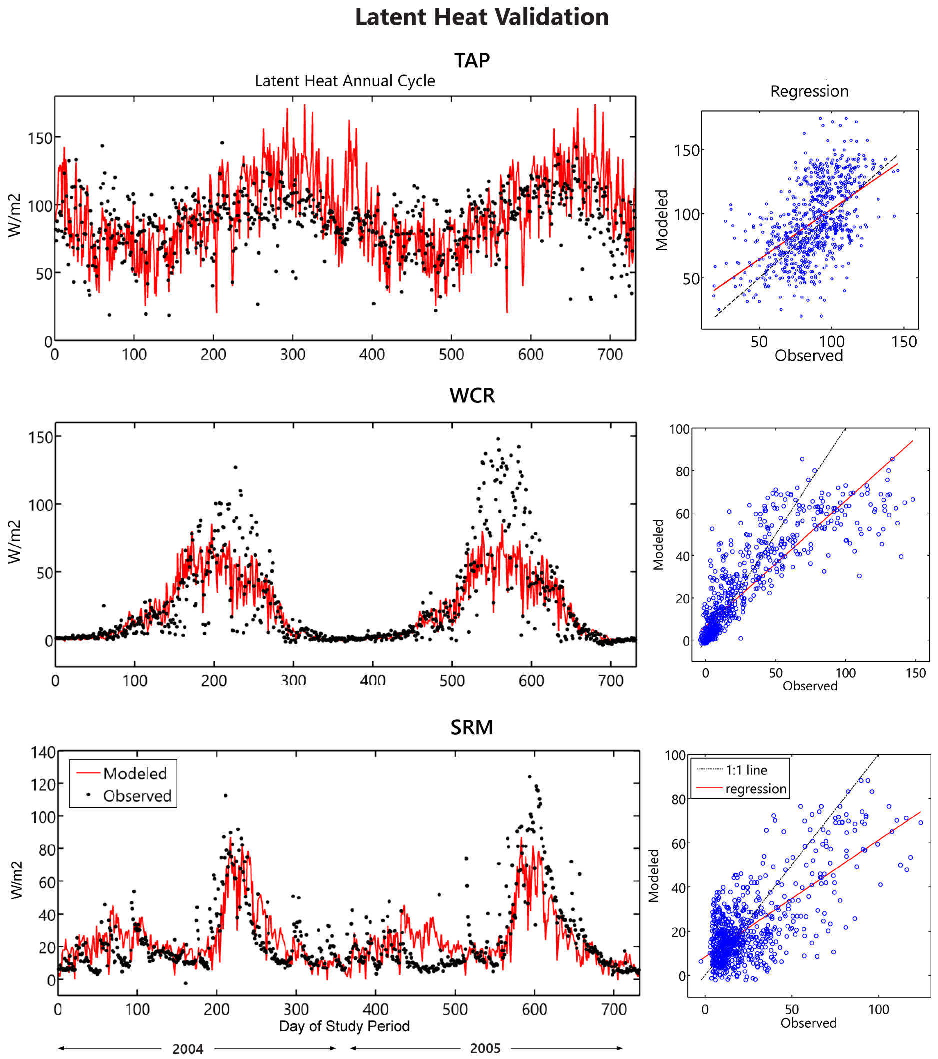

Figure S5. Latent heat validation for all sites.

Table S1: Functional group abbreviations for all sites

Abbrev.	WCR	SRM	TAP
UN	understory shrubs	understory shrubs	understory trees
MT	–	–	mid-canopy trees
OT	overstory trees	overstory trees	upper-canopy trees
L	–	–	lianas
MG	UN & OT	UN & OT	UN, MT, OT, & L

Sites: Willow Creek (WCR), Santa Rita Mesquite (SRM), and Tapajos National Forest (TAP). Multi-group scenarios (MG) include simulations for species interactions of all identified functional groups.

Table S2: List of Model Parameters

Site	WCR		SRM		TAP			
Functional Group	UN	OT	UN	OT	UN	MT	OT	L
Site Information								
Percent Sand[a]	63		75		10			
Percent Clay[a]	13		10		80			
Ecosystem Height[b] (m)	24		2.4375		32			
Flux Tower Observation Height[a] (m)	30		7.82		64			
Leaf Properties								
Vc_{max} (μmol/m^{2}s)	26.9[c]	44.8[d]	39[e]	17.62[e]	31.6[f]	57.5[f]	81[f]	59.7[f]
J_{max} (μmol/m^{2}s)	47.[c]	100[g]		13.55[e]	37.9[f]	81[f]	112[f]	87.5[f]
Respiration $Q10$ (mol/m^{2}s)	2.98[h]		3.36[m]		2[n]			
Root Properties								
Root Depth (m)	2.5	3	2.5	2.5	1	4	12	12
z_{50} [p]	0.19	0.2	0.24	0.28	.07	0.24	0.65	0.65
z_{95} [p]	1.71	1	0.65	1.5	0.4	1.5	4	4

[a] Fluxnet2015 Network[4–6]
[b] Modified from Fluxnet2015 Network[4–6] canopy heights based on leaf area density
[c] Nishida and Hanba[26]
[d] Racza et al.[27]
[e] Lee et al.[9]
[f] Domingues et al.[14]
[g] Inferred from Kubiske et al.[28]
[h] Bolstad et al.[29]
[m] Saito et al.[30]
[n] Melton et al.[31]
[p] Schenk and Jackson[32]
